# Right aortic arch analysis – Anatomical variant or serious vascular defect?

**DOI:** 10.1186/s12872-017-0536-z

**Published:** 2017-04-19

**Authors:** Agata Arazińska, Michał Polguj, Konrad Szymczyk, Magdalena Kaczmarska, Łukasz Trębiński, Ludomir Stefańczyk

**Affiliations:** 1 Department of Radiology, Medical University of Łódź, Kopcińskiego 22, 90-153 Łódź, Poland; 2 Department of Angiology, Interfaculty Chair of Anatomy and Histology, Medical University of Łódź, ul. Narutowicza 60, 90-136 Łódź, Poland

**Keywords:** Right aortic arch, Aberrant subclavian artery, Brachiocephalic trunk, Subclavian artery stenosis

## Abstract

**Background:**

The right-sided aortic arch (RAA) is a rare congenital defect of the aorta. The aim of the study was to assess the occurrence of RAA in diagnoses performed by the University Radiology Department and analyze the frequency of concomitant vascular abnormalities.

**Methods:**

The database of the Radiology Department was retrospectively analyzed between January 2008 and May 2016 with the keyword “right aortic arch”. Twenty patients with this diagnosis were identified from a total of 11,690 CT examinations of the chest area, 19,623 CT examinations of brain-supplying vessels, and 1863 MRI examinations of the heart and aortic arch or brain-supplying arteries. The type of aortic arch, the occurrence of Kommerell’s diverticulum and possible other vascular abnormalities, such as stenosis, kinking or occlusion, were then investigated.

**Results:**

The analysis identified nine patients with type I and 11 patients with type II RAA. Eight of the 11 type II patients presented Kommerell’s diverticulum. Concomitant vascular abnormalities were detected in four patients with type II RAA. In two cases, the right common carotid artery (RCCA) was narrowed by up to 80%, with steal phenomenon confirmed in one of them. In the second coincident right subclavian artery (RSA) stenosis was depicted. In two other cases, the aberrant left subclavian arteries (ALSA) were found to be narrowed at the level of origin by up to 70%. One patient was found with type B aortic dissection including ALSA and Kommerell’s diverticulum.

**Conclusions:**

Our observations indicate that concomitant vascular abnormalities may occur more often than reported in literature. Patients diagnosed with type II RAA should be examined with Doppler ultrasonography to identify coincident vascular disorders, especially stenosis of the common carotid arteries or subclavian arteries.

**Electronic supplementary material:**

The online version of this article (doi:10.1186/s12872-017-0536-z) contains supplementary material, which is available to authorized users.

## Background

A right-sided aortic arch is a rare congenital defect of the aorta. It is present in 0.05% to 0.1% of radiology series and in 0.04%-0.1% of autopsy series [1, 2]. Several classifications of these anomalies have been proposed based on the arrangement of the arch vessels, relationships with the esophagus, or the presence of congenital heart anomalies. The Edwards classification describes three types: RAA (right aortic arch) with ALSA (aberrant left subclavian artery), RAA with mirror image branching, and RAA with isolation of the left subclavian artery (LSA) [1, 2].

In RAA with ALSA, ALSA may arise from a remnant of the left dorsal aortic root (Kommerell’s diverticulum). This anomaly rarely produces symptoms and is usually an incidental radiological finding. Symptoms of esophageal compression may develop in older individuals with ectasia, tortuosity or aneurysm of the ALSA [3, 4]. Respiratory symptoms due to tracheal compression may be present in pediatric patients. This anomaly is rarely associated with other cardiovascular abnormalities. RAA with mirror image is usually associated with a form of cyanotic congenital heart disease, especially tetralogy of Fallot and truncus arteriosus. In RAA with isolation of the LSA, the LSA does not have a connection with the aorta, but is connected to the pulmonary artery by a left ductus arteriosus. This rare anomaly may cause congenital subclavian steal syndrome and vertebrobasilar insufficiency. It may be associated with congenital heart disease, especially tetralogy of Fallot [2, 3]. The aim of the present study was to review previous multidetector computed tomography (MDCT) angiography and magnetic resonance imaging findings of the right-sided aortic arch, which were additionally evaluated in ultrasonography, to determine the occurrence of concomitant vascular insufficiency of the aortic arch arteries in patients with RAA.

## Methods

The database of the Radiology Department was retrospectively analyzed between January 2008 and May 2016 using the keyword “right aortic arch”. This database comprised 11,690 CT examinations of the chest, 19,623 CT examination of brain-supplying vessels, and 1863 MRI examinations of the heart and aortic arch or brain-supplying arteries. Of these, 20 patients were identified.

The patients had undergone contrast-enhanced computed tomography angiography, slice thickness 0.625 mm-1.5mm (Light-Speed 64 VCT, GE, Waukesha, Wisconsin, US) and/or magnetic resonance angiography. Magnetic resonance angiography was performed using time-of-flight technique and after administering a contrast agent (Magnetom Avanto 1.5 T, Siemens, Erlangen, Germany). Table 1 presents a summary of the examinations of the patients. Consecutive examinations were evaluated using the software available at the Radiology Department (Advantage Workstation 4.4 GE Waukesha, Wisconsin, US), allowing cases with right-sided aortic arch to be identified. Computed tomography and MRI scans were performed with subsequent sagittal, coronal and 3-D reformations. The analysis included a review of the occurrence of types of RAA, the presence of Kommerell’s diverticulum and concomitant status of aortic arch arteries (hypoplasia, kinking, stenosis and occlusion), the occurrence and degree of stenosis of the vessels originating from the aortic arch in response to anatomical structures, arteriosclerosis or the presence of such concomitant complications as thrombosis or dissection.

**Table 1:** Performed examination in studied patients

Types of right aortic arch found in studied patients	Types of performed examinations			
	MRI	CTA	MR + CT	Doppler ultrasound
I	6	0	3	9
II	0	10	1	11

I –type of right aortic arch with mirror image branching; II –type of right aortic arch with aberrant left subclavian artery; MRI – magnetic resonance imaging; CTA – computed tomography angiography

Visual assessment of all examinations was performed by two study readers. Identified records were retrospectively reviewed by the investigator conducting the database analysis and the investigator who performed the initial examination of the patient (collected in the database). No discrepancies were found between the analyses of the study readers.

In all cases, evaluation with Doppler ultrasound was performed by a radiologist with over 10 years of experience. An ultrasound examination of aortic arch arteries (subclavian, carotid and vertebral) was performed using a 6-15 MHz linear probe (Logiq 7, GE, Waukesha, Wisconsin, US). In the studied cases, the spectral Doppler waveform was assessed in the vessels of the aortic arch, common carotid arteries, vertebral arteries, and subclavian arteries. Evaluation was performed in accordance with the guidelines of the Society of Radiologists in Ultrasound [5]. Our study was conducted with the approval of the local ethical committee of the Medical University of Lodz (approval number: RNN/259/15/KE).

## Results

The 20 cases of right-sided aortic arch included eight women and 12 men aged between 11 and 76, mean age 43.50.

No significant stenosis or aortic arch vessel occlusion was observed in the group of patients with RAA type I. This was confirmed by the presence of symmetrical velocity spectra in Doppler examinations: most of the patients had undergone cardiosurgical procedures due to congenital heart disorders.

**Fig. 1:**
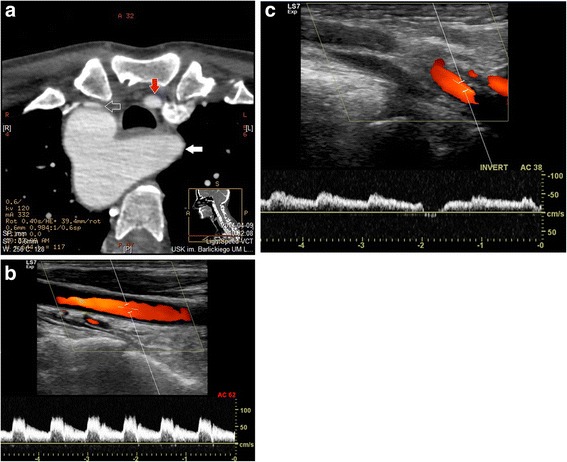
**a** Computed Tomography Angiography of the aortic arch, transverse scan of 51 y.o. woman with several stenoses of the right common carotid artery - compression between the aortic arch and sternoclavicular joint (gray arrow). The left common carotid artery has a normal lumen (*red arrow*). The left subclavian artery in aberrant configuration (*white arrow*). **b** Doppler ultrasound Spectrum of left common carotid artery confirmed typical flow Systolic velocity about 80 cm/s diastolic velocity 35 cm/s. **c** Doppler ultrasound Spectrum of right common carotid artery confirming severe stenosis in the proximal part of the artery. Systolic velocity is low, about 45 cm/s, diastolic velocity 35 cm/s

**Fig. 2:**
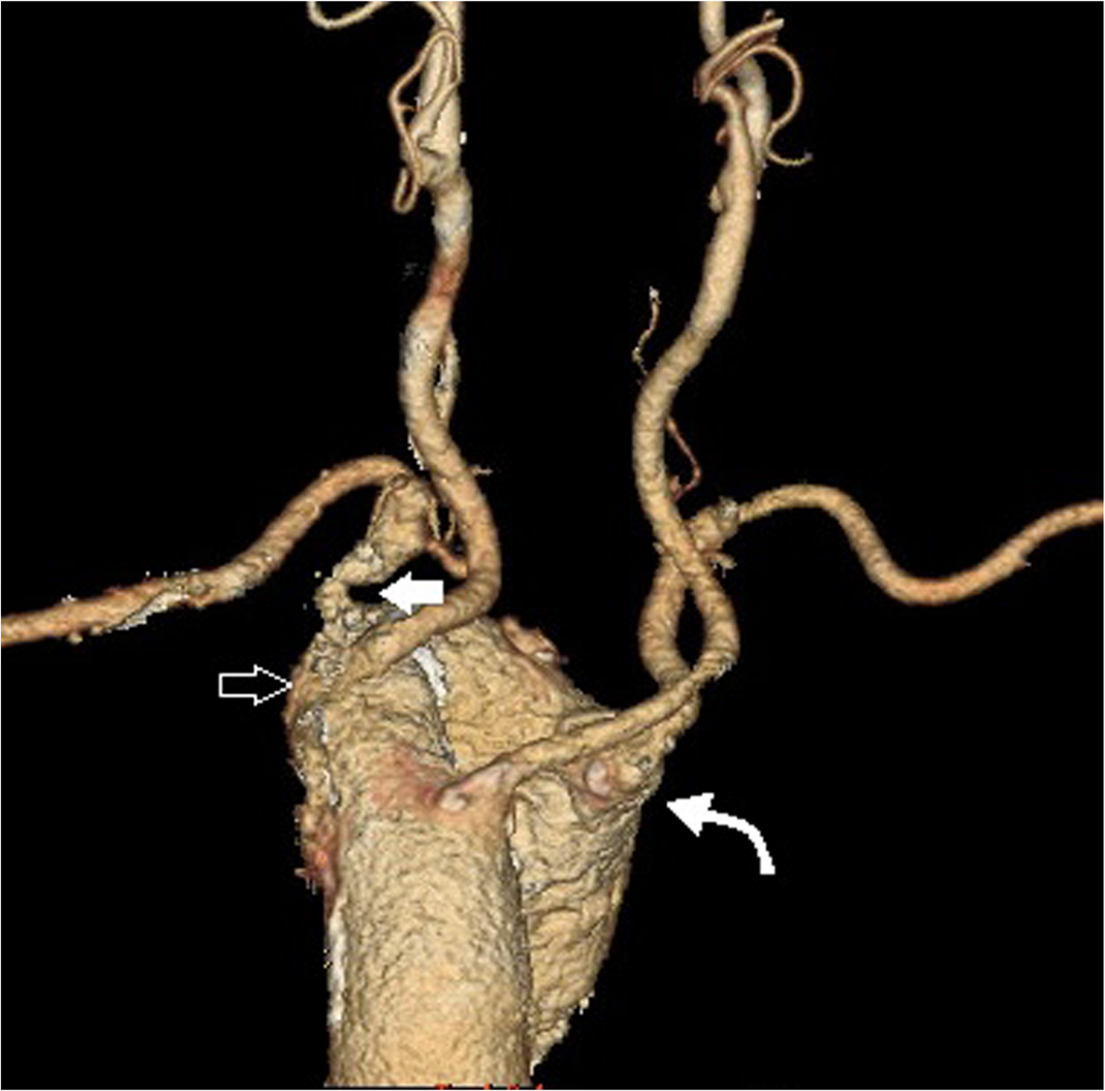
Computed tomographic angiography of the aorta, volume rendering of the right-sided aortic arch. Vessels in the following order: left common carotid artery (*without arrow*), right common carotid artery, stenotic up to 80% (*right-pointing arrow*), narrowed origin of the right subclavian artery with post-stenotic widening (left-pointing arrow), aberrant left subclavian artery with bulbous origin (*curved arrow*)

**Fig. 3:**
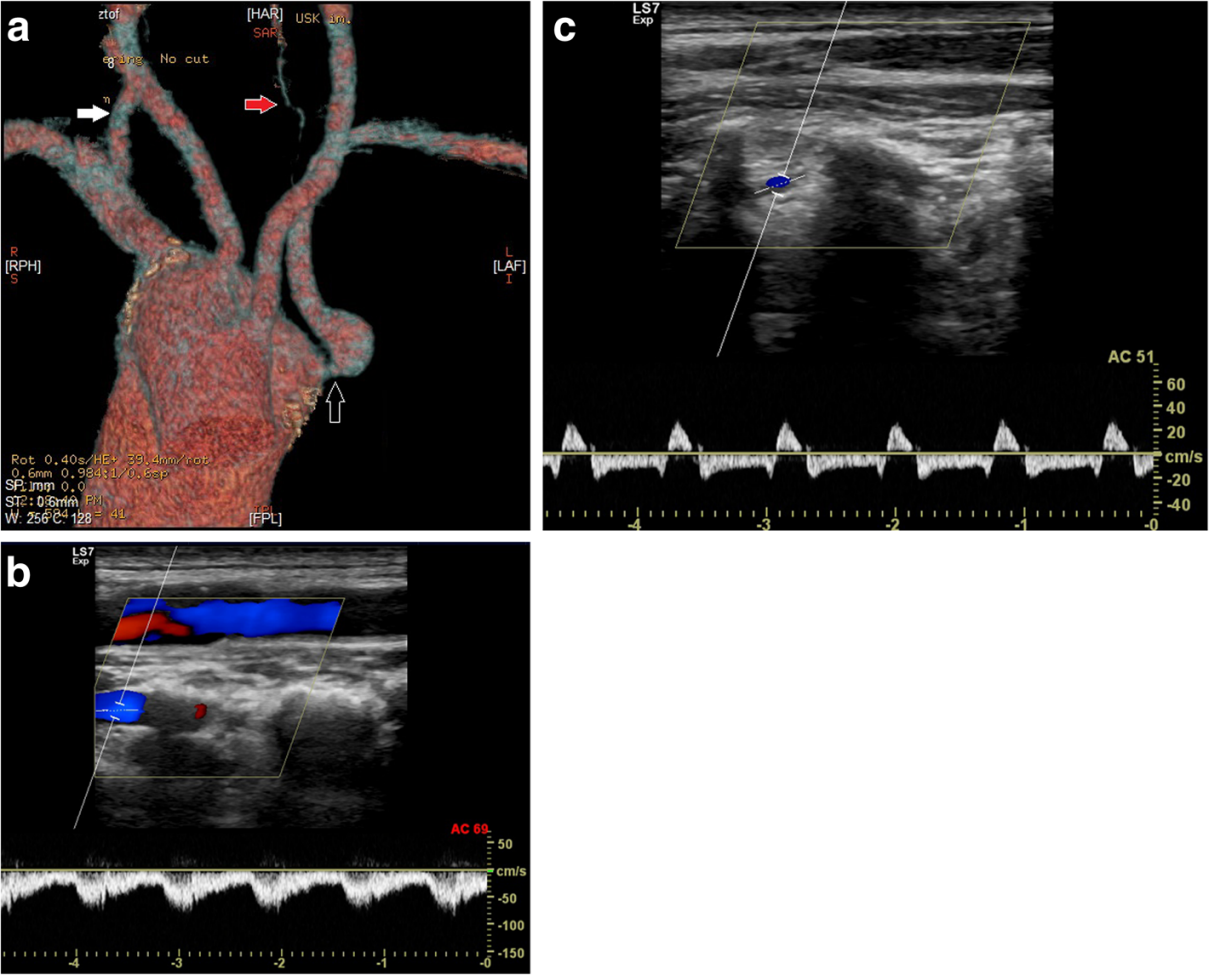
**a** Thoracic computed tomography, volume rendering, of right-sided aortic arch with vessels in the following order: Left common carotid artery, Right common carotid artery, Right subclavian artery, Aberrant left subclavian artery. Stenotic origin of ALSA with post-stenotic widening (*Black arrow*), right vertebral artery (*white arrow*), hypoplastic left vertebral artery (*red arrow*). **b** Doppler ultrasound Spectrum of right vertebral artery confirmed typical flow. Systolic velocity about 70 cm/s, diastolic velocity 40 cm/s. **c** Doppler ultrasound Spectrum of left vertebral artery confirmed severe stenosis in the proximal part of the artery. Typical flow for steal syndrome (bidirectional)

In the group of patients with RAA type II, two patients (women, aged 51 and 76) were diagnosed with stenosis of a vessel caused by anatomical compression. Significant compressions were depicted at the site of departure of RCCA, between the aortic arch and the sternoclavicular joint. The RCCAs of both patients were narrowed up to 80% (Fig. 1a-c). In both patients, right-sided aortic arch with self-departing carotid and subclavian arteries was found. However, both patients present an aberrant left subclavian artery. In both cases, the origin of ALSA was also found to have a bulbous configuration (Kommerell’s diverticulum), and one of the patients was also found to have RSA non-significant stenosis at the origin of vessels with poststenotic widening (Fig. 2). In another two cases, LSAs, which were found to be lusorian arteries, were narrowed by up to 70% at the place of origin with poststenotic widening (Fig. 3a-c). One male patient was diagnosed with type B aortic dissection covering the left subclavian artery. The examination revealed anomalies in the departure of vessels from the aortic arch: an aberrant left subclavian artery, concomitant self-departing carotid arteries and innominate artery were present.

In our group, no patients with type III RAA were identified.

No significant stenosis of the aortic arch arteries caused by arteriosclerotic changes or thrombosis was present. The results are presented in Table 2.

**Table 2:** Results

Types of right aortic arch found in studied patients	Number of patients (%)	Gender	Range of age (years)	Arch artery anomalies			
				Kommerell’s diverticulum	Compression and stenosis	Kinking and stenosis	dissection
I	9 (45%)	4F, 5 M	10-74	0	0	0	0
II	11 (55%)	4F, 7 M	19-76	8	2 (RCCA)	2 (RSA)	1 (RSA)

I –type of right aortic arch with mirror image branching; II –type of right aortic arch with aberrant left subclavian artery; RCCA –right common carotid artery; RSA –right subclavian artery; F – female; M - male

## Discussion

During the growth of the embryo, six pairs of aortic arches develop at different stages of organogenesis. While the left fourth primitive aortic arch forms the adult aortic arch, the right fourth generally disappears, resulting in the normal course of the aorta arching to the left and descending to the left of the spine; however, if the left fourth arch disappears and the right persists, a right aortic arch develops. If both arches persist, they form a double arch or a vascular ring encircling the trachea and esophagus. Numerous classifications of these anomalies have been described, based on the arrangement of aortic arch vessels, the relationships with the esophagus, or the presence of congenital heart anomalies. Depending on the author, Type I is reported to represent up to 59% of all right aortic arches (in most of the literature it is reported to be less common than type II), type II 39.5%, and type III 0.8%. In RAA with ALSA (type II) the first branch arising from the aortic arch is the left carotid artery, which is followed by the right carotid artery, right subclavian arteries and ALSA. In RAA with a mirror image, the left innominate artery is the first branch arising from the arch, which is followed by the right carotid artery and right subclavian arteries. In RAA with isolation of the LSA, the left carotid artery arises as the first branch of the right arch, followed by the right carotid artery and right subclavian arteries. The LSA does not have a connection with the aorta, but it is connected with the pulmonary artery by a left ductus arteriosus [1, 2, 6, 7].

Congenital heart anomalies such as Tetralogy of Fallot (ToF), pulmonary stenosis with ventricular septal defects, tricuspid atresia, and truncus arteriosus are present in 75% to 85% of type I and type III RAA, and in 5% to 10% of type II RAA [1]. In accordance with literature reviews, our patients diagnosed with mirror-image type RAA (9/20) also have a positive medical history for ToF (5/9).

In our study the most common type of vessel branching (11/20 patients) was found to be type II RAA. This type of RAA is reported to rarely produce symptoms. In infancy, symptoms of this type of RAA are related to congenital heart anomalies or to compression of mediastinal structures such as the trachea or the esophagus. No congenital heart anomalies were found in our group of patients with type II RAA (RAA with ALSA). In adulthood, symptoms associated with this type of RAA are more often the result of early atherosclerotic changes of anomalous vessels, as 5% of adult patients with an aberrant subclavian artery have symptoms associated with the development of atherosclerosis [8], dissection, or aneurysmal dilatation accompanied by compression of surrounding structures; this causes dysphagia (dysphagia lusoria—dysphagia by a trick of nature), dyspnea, stridor, wheezing, cough, choking spells, recurrent pneumonia, obstructive emphysema, or chest pain [4]. The aberrant subclavian artery may be located behind the esophagus (80%), between the esophagus and trachea (15%) or in front of the trachea (5%), and can cause symptoms even in the absence of an aneurysm [1, 4, 9].

The development of aneurysm usually occurs at the level of origin of an aberrant left subclavian artery and is known as Kommerell’s aneurysm. In the studied group, eight of the 11 patients with type II of RAA were found to have Kommerell’s diverticulum. Literature reviews have found Kommerell’s diverticulum to be presented by up to 100% of patients with a right aortic arch with an aberrant left subclavian artery (RAALS) diagnosed by routine CT examination [10]. Most patients with Kommerell’s diverticulum are asymptomatic unless aneurysmal disease develops. Recent histopathological studies have revealed a high prevalence of cystic medial necrosis in the Kommerell’s diverticulum wall, potentially accounting for the increased risk of Kommerell’s diverticulum dissection and rupture [11]. Despite its rarity, this condition is clinically relevant because of the mortality associated with rupture, the morbidity caused by compression of mediastinal structures, and the complexity of surgery [1, 2].

Complications associated with a kink or stenosis of the subclavian artery, or aortic pseudocoarctation are less commonly observed [12]. In the present study, four patients with such abnormalities were detected: two of whom, both women with type II RAA, presented RCCAs which were narrowed by up to 80% at the place of origin, between the aortic arch and sternoclavicular joint. Moreover, one of these patients presented a concomitant stenosis of the RSA by up to 50% at the level of its departure. In both cases, Kommerell’s diverticulum was present. In one of the presented cases the indication for examination was the presence of discrete cerebrovascular insufficiency symptoms which had been increasing in severity for about 2 years. Further examination (Doppler ultrasonography, angio-MRI) revealed steal phenomenon increasing on exertion due to reversal of the pressure gradient between the arterial circle of the brain and the RCCA, resulting in periodic reversed flow in the RICA.

Another two patients (one woman, one man) were diagnosed with ALSA stenosis of 70% at the level of its origin with poststenotic widening. In literature, such cases are rarely described, but they are nevertheless clinically significant [12–14]. One of the cited cases demonstrates that a right-sided aortic arch with an aberrant subclavian artery can be diagnosed prenatally, and that stenosis of the subclavian artery can occur in early infancy [13]; the neonatologist or pediatrician should be aware of this, and that stent implantation represents a minimally-invasive therapeutic approach.

In our study, one patient (male) was diagnosed with type B aortic dissection which included also ALSA, all mesenteric arteries and passed through common and external iliac arteries. In addition, a rare configuration of arteries departing from aortic arch was observed: the left common carotid artery, right common carotid artery, innominate artery and left subclavian artery originated from the proximal aorta in said order. In a literature review of aneurysm associated with right-sided arch, 6% of affected patients presented with rupture and 53% with either rupture or dissection. The size at which these aneurysms rupture cannot be predicted because of the relative rarity of the condition and the limited information available [1]. The most severe issues in the course of aneurysm are its higher propensity towards acute rupture with increasing aneurysm size, and the risk of hemodynamic impairment with distal hypoperfusion or embolization due to thrombosis of the aneurysm lumen, which is particularly probable in the case of aneurysms of the supra-aortic vessels. Clinical and imaging diagnoses focus on the reduction of morbidity and mortality associated with such risks, yet aneurysmal disease may often go undetected due to lack of symptoms, unless imaging procedures of the upper chest are performed for diagnosis or follow-up of concurrent disease [11].

The study has some limitations regarding the study group. It was quite small, including only 20 patients with RAA; however, more patients are being gathered to continue the study. In addition, the sample is also quite heterogeneous with regard to patients’ age, the reasons for which examinations were performed and diagnoses; there is also a lack of data from patients with type III RAA, with the majority of patients presenting type II RAA, which is rarely associated with congenital heart diseases and rarely produces symptoms. In addition, four patients were found to possess concomitant vascular abnormalities that may be clinically important; our observations indicate that concomitant vascular abnormalities may occur more often than literature reports might suggest.

It is important to highlight the fact that despite being a rather uncommon anomaly, patients with RAA should be carefully examined for coincident vascular defects and their possible complications, particularly in elderly patients. Aging is an important determinant of cardiovascular risk and is associated with numerous changes in the structure and functioning of the cardiovascular system including the large arteries and vertebrobasilar system [15]. With age, the aorta stiffens, dilates and becomes tortuous. Hicson et al. [16] report the greatest difference in systolic diameter and length in a group of old and young subjects to be in the aortic arch: this may be compensatory to some extent, to maintain capacitance in the face of increased wall stiffness. Therefore, thorough scrutiny is essential in elderly patients presenting symptoms related to anomalous vessels which are intensified by atherosclerotic changes. The use of Doppler ultrasound is recommended to assess any possible impairment of patency present in most situations, apart from widening. Although no patients with type III RAA are included in the present group, we are convinced that this method would be a suitable diagnostic tool in this group. It has been found to be effective in cases of asymptomatic vascular anomalies: subclavian steal phenomenon was detected in one asymptomatic patient in the course of thrombosis of Kommerell’s diverticulum, extending to the prevertebral tract of an aberrant right subclavian artery [11].

## Conclusion

The occurrence of type II RAA may coincide with that of related, clinically important vascular abnormalities, particularly the compression or kinking of vessels with significant stenosis. Despite the presence of non-specific clinical symptoms, uncommon arterial variations could be important for diagnostic issues. In this situation, long-term Doppler ultrasound monitoring may well be a valuable diagnostic method.

## Additional file

Additional file 1: Material data. (XLSX 9 kb)

## Abbreviations

ALSA: Aberrant left subclavian arteries
LSA: Left subclavian artery
MDCT: Multidetector computed tomography
RAA: Right-sided aortic arch
TAR: Total arch replacement
